# Supplementary material for: Aβ(1-42) tetramer and octamer structures reveal edge conductivity pores as a mechanism for membrane damage
Source: Nat Commun. 2020 Jun 15;11:3014. doi: 10.1038/s41467-020-16566-1 (PMC7296003; doi:10.1038/s41467-020-16566-1)
Supplement: Supplementary file 1 — Supplementary Information [file 41467_2020_16566_MOESM1_ESM.pdf]

Supplementary Information

Aβ(1-42) tetramer and octamer structures reveal edge conductivity pores as a mechanism for membrane damage

Sonia Ciudad, Eduard Puig, *et al.*

This PDF file contains:

- Supplementary Figures 1 – 27
- Supplementary Tables 1 – 5
- Supplementary References

Supplementary Figures

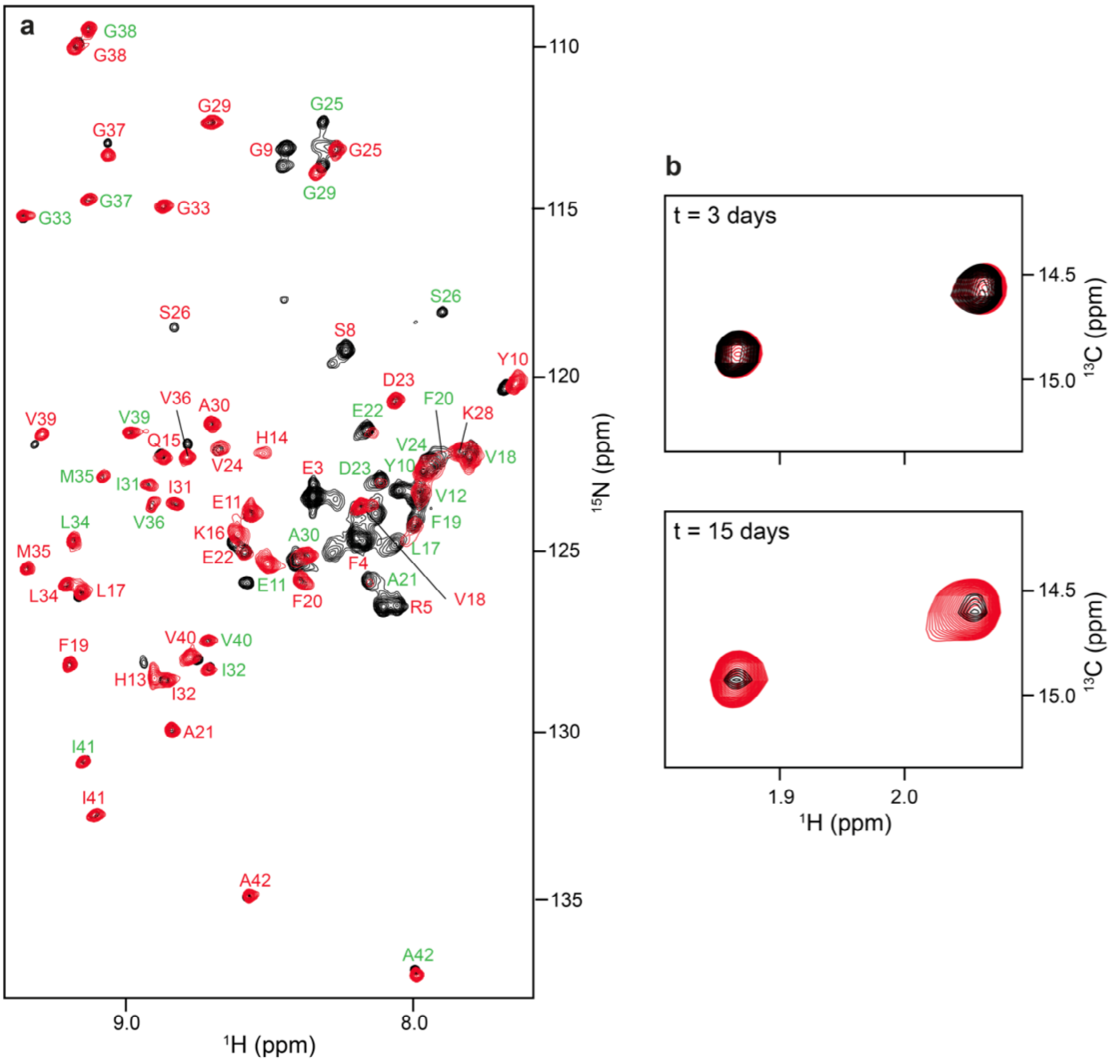

Supplementary Fig. 1. βPFOs$_{Aβ(1-42)}$ form at pH 7.4 and at pH 9.0. (**a**) Overlays of ^{1}H-^{15}N TROSY-HSQC NMR spectra of ^{2}H,^{15}N βPFOs$_{Aβ42}$ prepared at pH 7.4 (black) and at pH 9.0 (red). (**b**) Overlay of ^{1}H-^{13}C HMQC NMR spectra of Met^{35}-[$^{13}CH_3$] βPFOs$_{Aβ42}$ prepared at pH 7.4 (black) and at pH 9.0 (red) after 3 days incubation (top) and 15 days incubation (bottom).

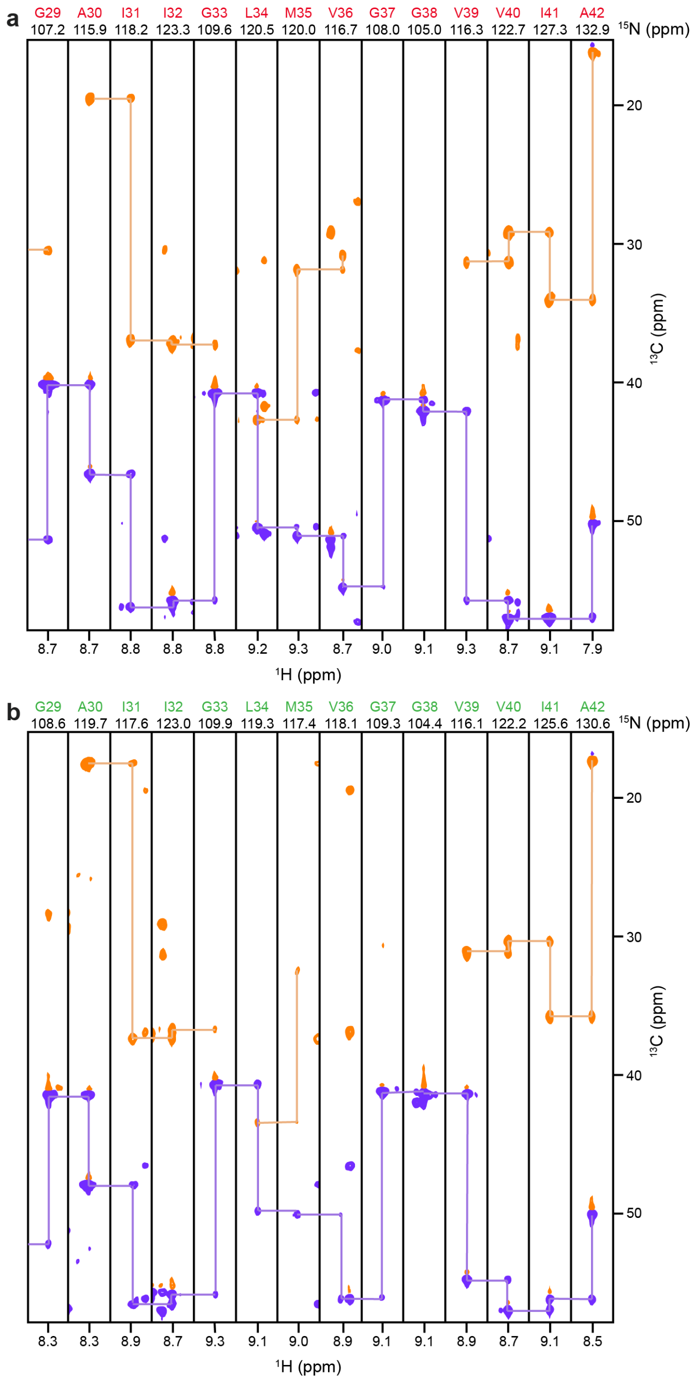

Supplementary Fig. 2. Sequential assignments for $\beta PFOs_{A\beta(1\text{-}42)}$ sample. Strips for residues 29-42 for (**a**) red Aβ(1-42) subunit and (**b**) green Aβ(1-42) subunit from a 3D TROSY-HNCACB experiment obtained using a $^{2}H,^{15}N,^{13}C$ $\beta PFOs_{A\beta(1\text{-}42)}$ sample. Purple and orange cross-peaks arise from Cα and Cβ nuclei, respectively. The purple and orange lines indicate the sequential connection between the strips.

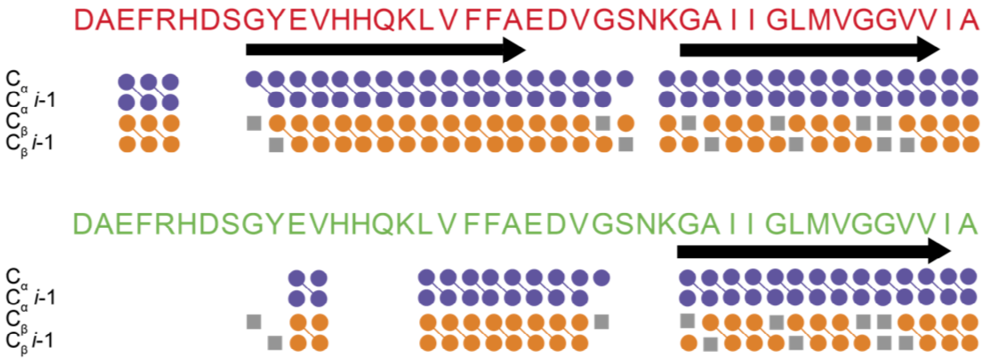

Supplementary Fig. 3. Sequence-specific resonance assignments of $\beta PFOs_{A\beta(1-42)}$ sample. Residues for which the intra-residual Cα, sequential Cα, intra-residual Cβ and sequential Cβ were observed in the 3D TROSY-HNCA or the 3D TROSY-HNCACB are marked, respectively, as a dot in the rows CA, CA_{i-1}, CB, CB_{i-1}. Lines indicate connectivity between the atoms. Gray boxes indicate unobservable signals (no Cβ for Glycine). The arrows show the location of the three β-strands.

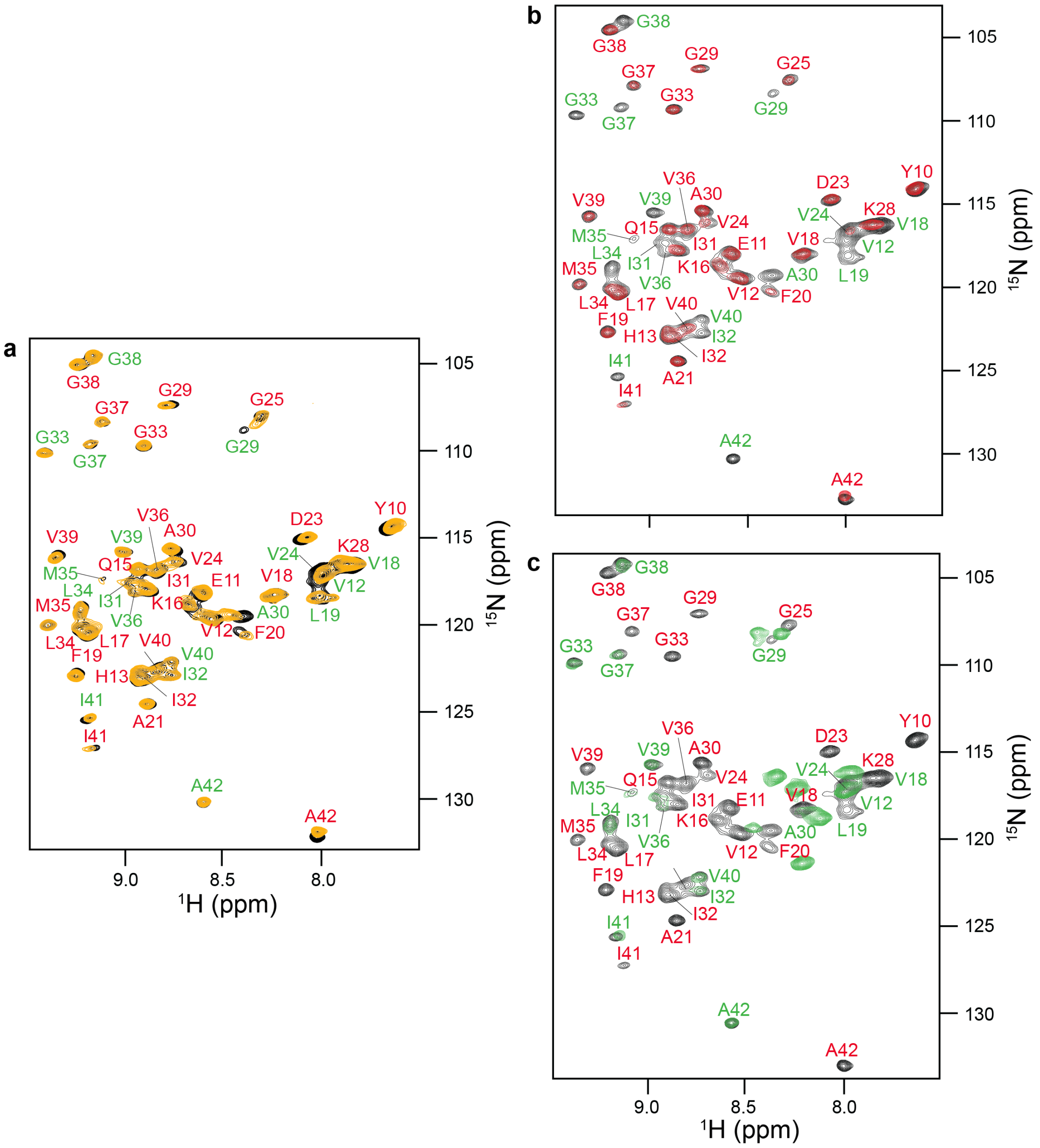

Supplementary Fig. 4. Secondary structural elements connectivity within βPFOs$_{A\beta(1-42)}$ sample. 2D [^{1}H,^{15}N]-HSQC spectra of mixtures of Aβ(1-42) and Aβ(17-42) with different labeling schemes to unambiguously establish the connectivity between β1 and β2, and α1 and β3 secondary structural elements. We hypothesized that a sample prepared using a mixture of Aβ(1-42) and Aβ(17-42) should form the same tetramer structure as Aβ(1-42) by itself, since Aβ(1-42) and Aβ(17-42) comprise, respectively, the required residues to incorporate in the tetramer as the red and green Aβ subunits.

(**a**) We confirmed this hypothesis by establishing that the 2D [^{1}H,^{15}N]-HSQC spectrum of a sample prepared using a mixture of ^{15}N Aβ(1-42) and ^{15}N Aβ(17-42) in orange, was identical to that of a sample prepared using ^{15}N Aβ(1-42) alone, shown in black. (**b**) Next, we measured 2D [^{1}H,^{15}N]-HSQC spectrum of a tetramer sample prepared using a mixture of ^{15}N Aβ(1-42) and ^{14}N Aβ(17-42) shown in red and compared it to that obtained for a sample prepared using only ^{15}N Aβ(1-42) in black. Only residues assigned to β1 and β2 of the red Aβ subunit were detected. (**c**) Finally, we measured 2D [^{1}H,^{15}N]-HSQC spectrum of a tetramer sample prepared using a mixture of ^{14}N Aβ(1-42) and ^{15}N Aβ(17-42) shown in green and compared to that obtained for a sample prepared using only ^{15}N Aβ(1-42) in black. Only residues assigned to α1 and β3 of the green Aβ subunit were observed. In summary, these results validated our assignments, providing conclusive evidence for β1 being connected to β2 within the red Aβ(1-42) subunit and α1 being connected to β3 within the green Aβ(1-42) subunit, thereby further confirming the Aβ(1-42) tetramer topology. In all 2D [^{1}H,^{15}N]-HSQC spectra, two Aβ(1-42) subunits were detected, and residues belonging to each of them were labeled in either red or green.

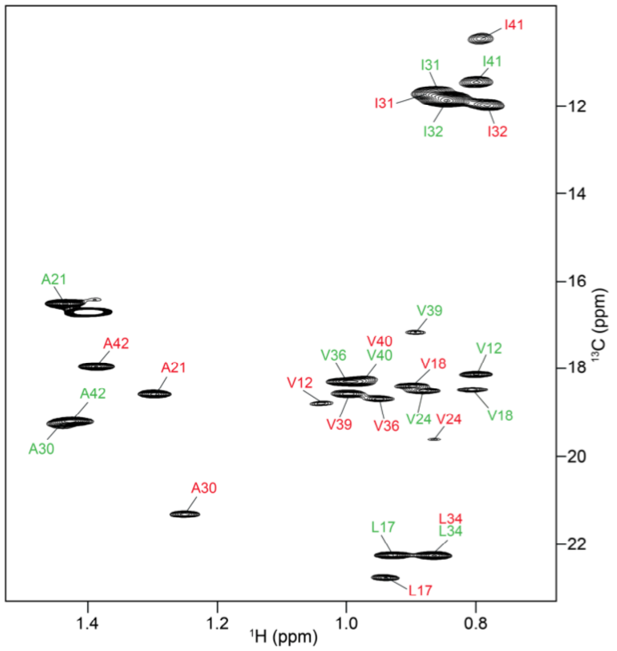

Supplementary Fig. 5. Alanine, isoleucine, leucine, and valine (AILV) methyl group resonance assignments of the Aβ(1-42) tetramer. The 2D [^{1}H,^{13}C]-TROSY spectrum of the Aβ(1-42) tetramer prepared using selectively ^{13}C methyl-protonated AILV and otherwise uniform ^{2}H,^{15}N Aβ(1-42) in DPC at 37°C. Two Aβ(1-42) subunits are detected and residues belonging to each of them are labeled in either red or green. All available resonance assignments are indicated (6/8 Ala, 6/6 Ile, 4/4 Leu, and 12/12 Val).

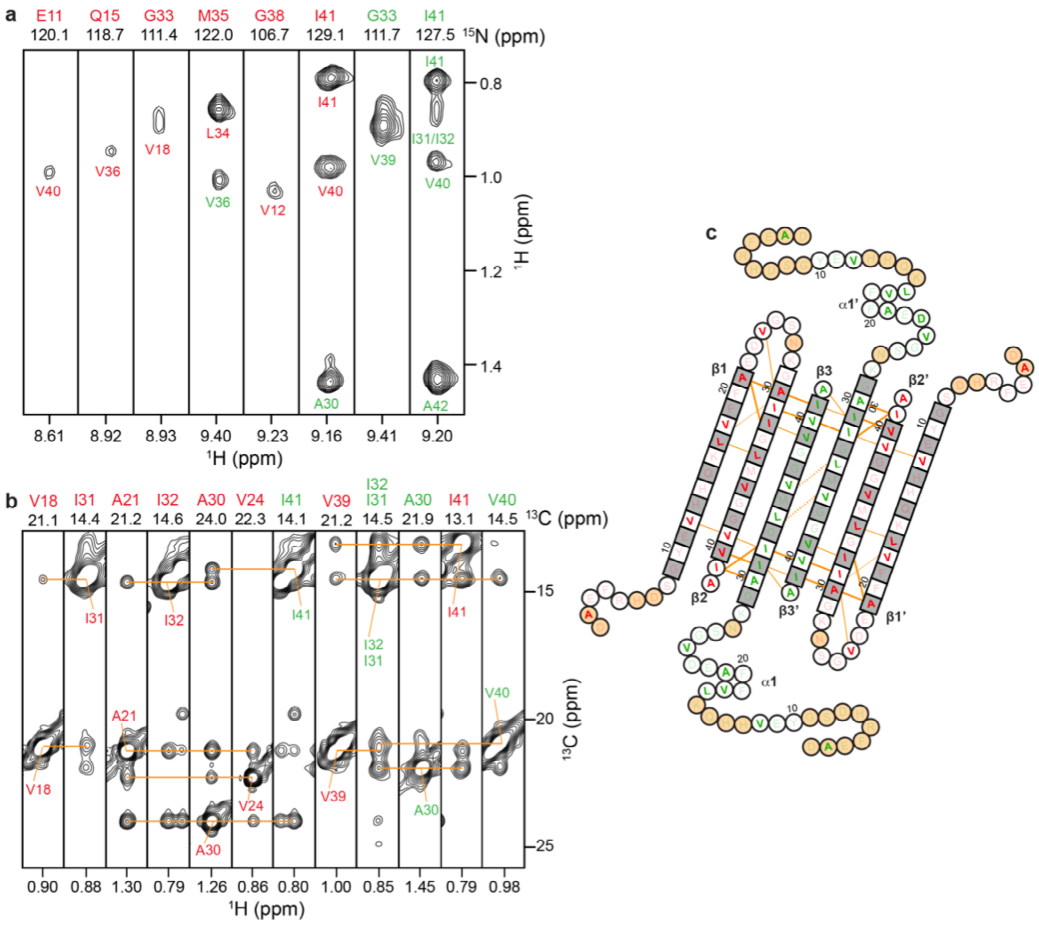

Supplementary Fig. 6. AILV methyl labeling to validate Aβ(1-42) tetramer topology. (**a**) NH-CH_3 NOE strips from a 3D NH-CH_3 NOESY spectrum and (**b**) CH_3-CH_3 NOE strips from a 3D CH_3-CH_3 NOESY spectrum. Both spectra were recorded using the Aβ(1-42) tetramer prepared using selectively ^{13}C methyl-protonated AILV and otherwise uniform ^{2}H,^{15}N Aβ(1-42) in DPC at 37°C. (**c**) Aβ(1-42) tetramer topology. The color of the amino acid indicates whether it belongs to the red or green Aβ(1-42) subunit and ^{13}C methyl-protonated AILV residues are highlighted. Amino acids in square denote β-sheet secondary structure, as identified by secondary chemical shifts; all other amino acids are in circles. Orange lines denote experimentally observed CH_3-CH_3 NOEs. For clarity, NH-CH_3 NOEs are not shown.

Bold lines indicate strong NOEs typically observed between hydrogen-bonded residues in β-sheets. Dashed lines show probable contacts between protons with degenerate ^{1}H chemical shifts. The side chains of white and grey residues point towards distinct sides of the β-sheet plane, respectively. Orange circles correspond to residues that could not be assigned.

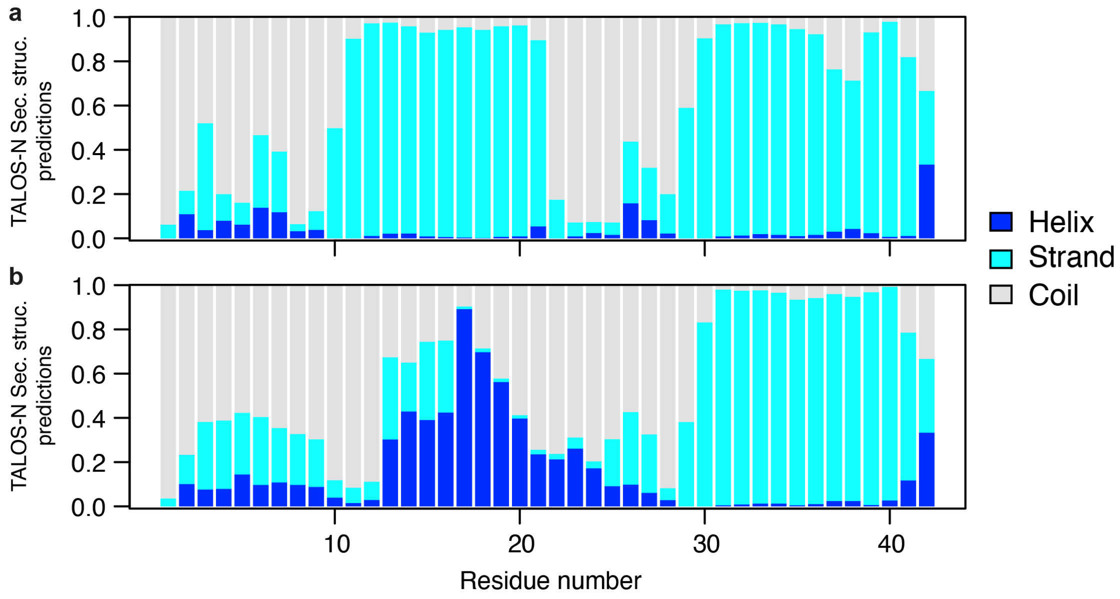

Supplementary Fig. 7. Secondary structure prediction confidence for Aβ(1-42) tetramer. Reported by TALOS+ from chemical shifts (N, Cα, C, Cβ and HN) for (**a**) The Aβ(1-42) red subunit and (**b**) the Aβ(1-42) green subunit that comprise the Aβ(1-42) tetramer.

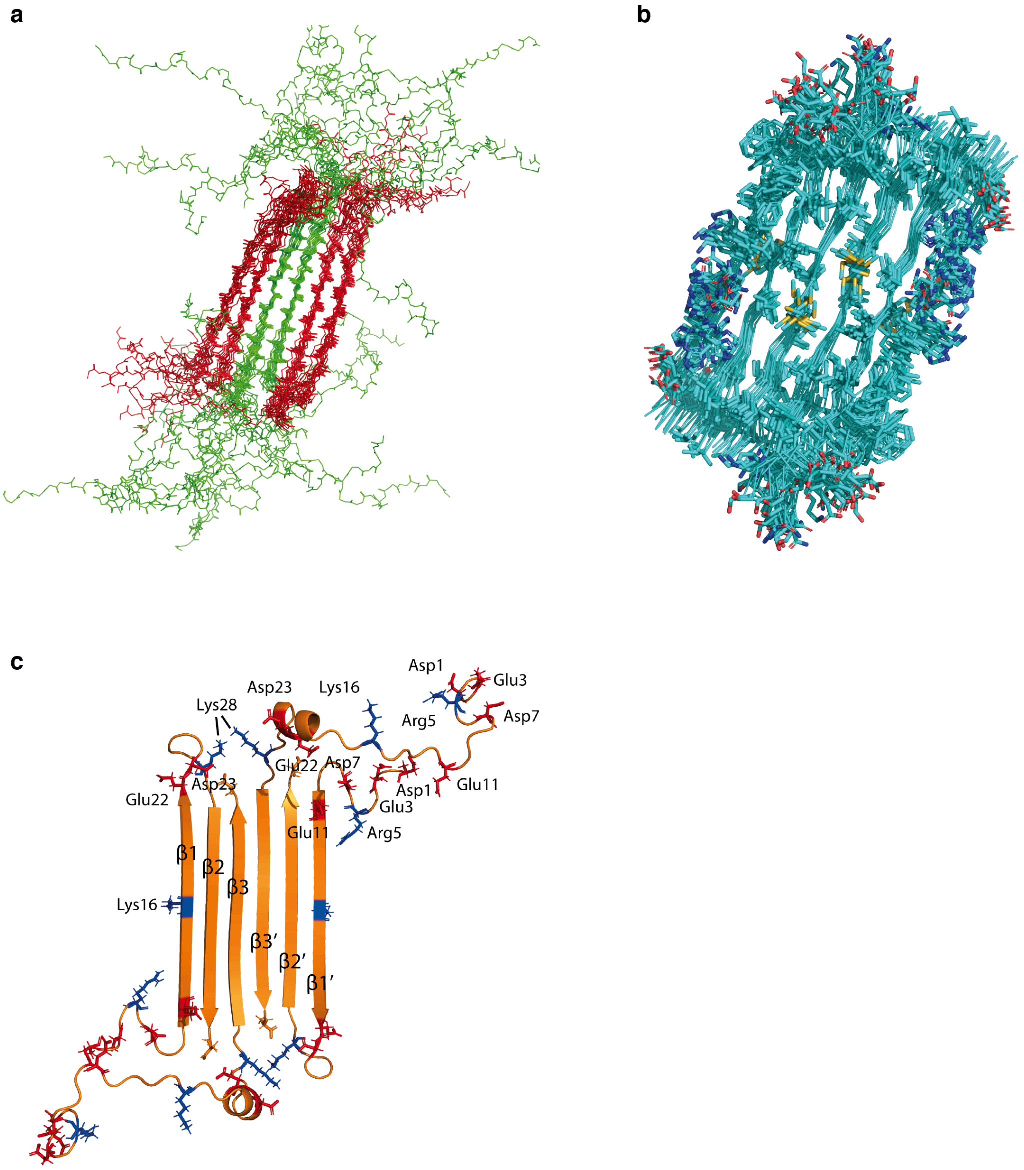

Supplementary Fig. 8. 3D structure of the Aβ(1-42) tetramer. NMR ensemble of the 10 lowest energy structures calculated for the Aβ(1-42) tetramer depicting (**a**) all backbone atoms and (**b**) backbone and side chain atoms of the β-sheet core. Residues in the six-stranded β-sheet core are used for superposition. (**c**) Aβ(1-42) tetramer with acidic and basic residues highlighted in red (aspartic, glutamic acid, C(t)) and blue (N(t), lysine, arginine), respectively. The representation shows the proximity of acid and basic residues as potential sites for zero-length cross-linking.

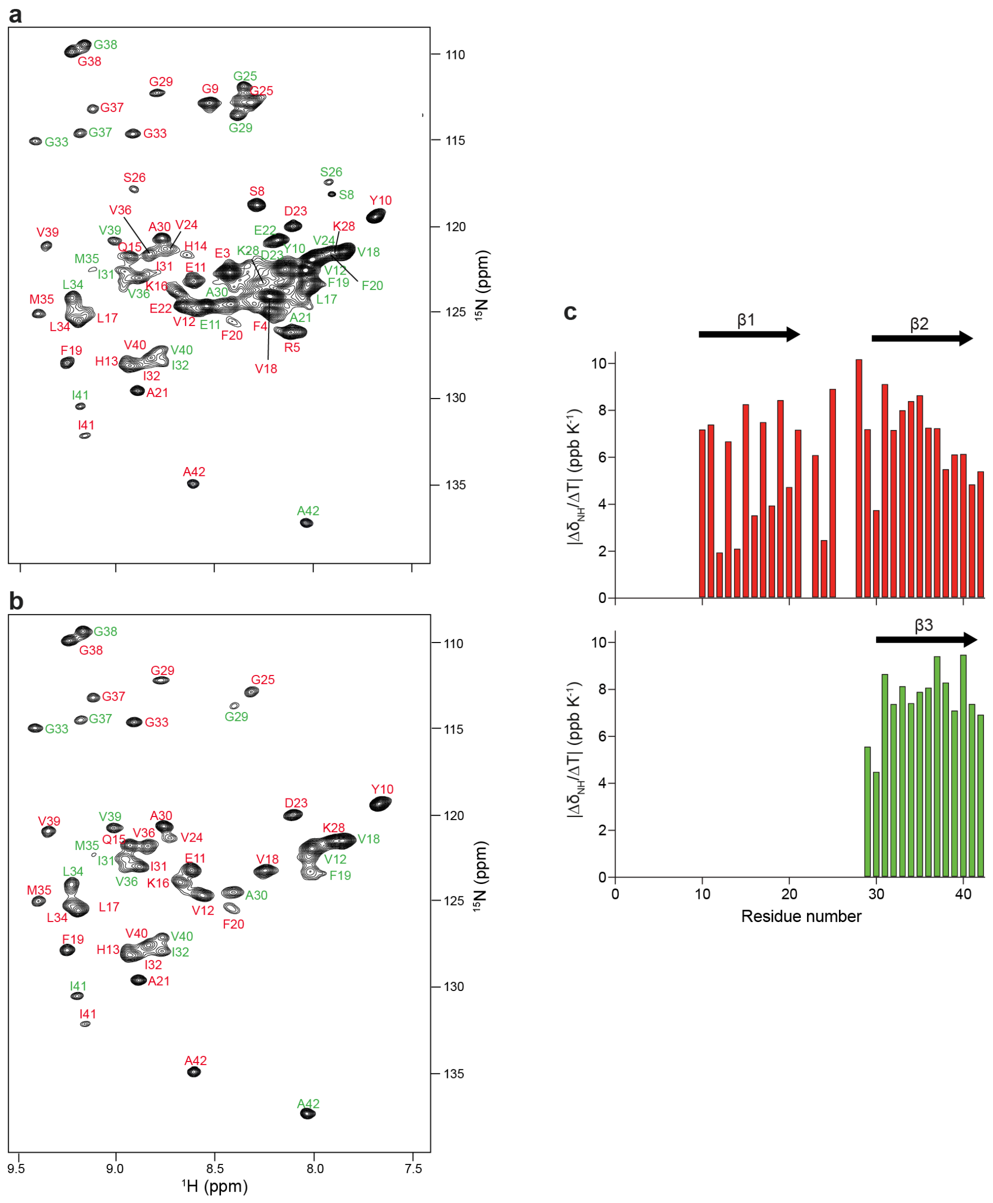

Supplementary Fig. 9. Water accessibility of amide protons of the Aβ(1-42) tetramer. 2D [^{1}H,^{15}N]-HSQC spectra obtained at (**a**) pH 8.5 and (**b**) pH 9.5. (**c**) Amide proton temperature coefficients versus residue number for the red (top) and the green (bottom) Aβ(1-42) subunits. Secondary structural elements are shown at the top with their corresponding number. Arrows indicate β-strands and helical symbols helices.

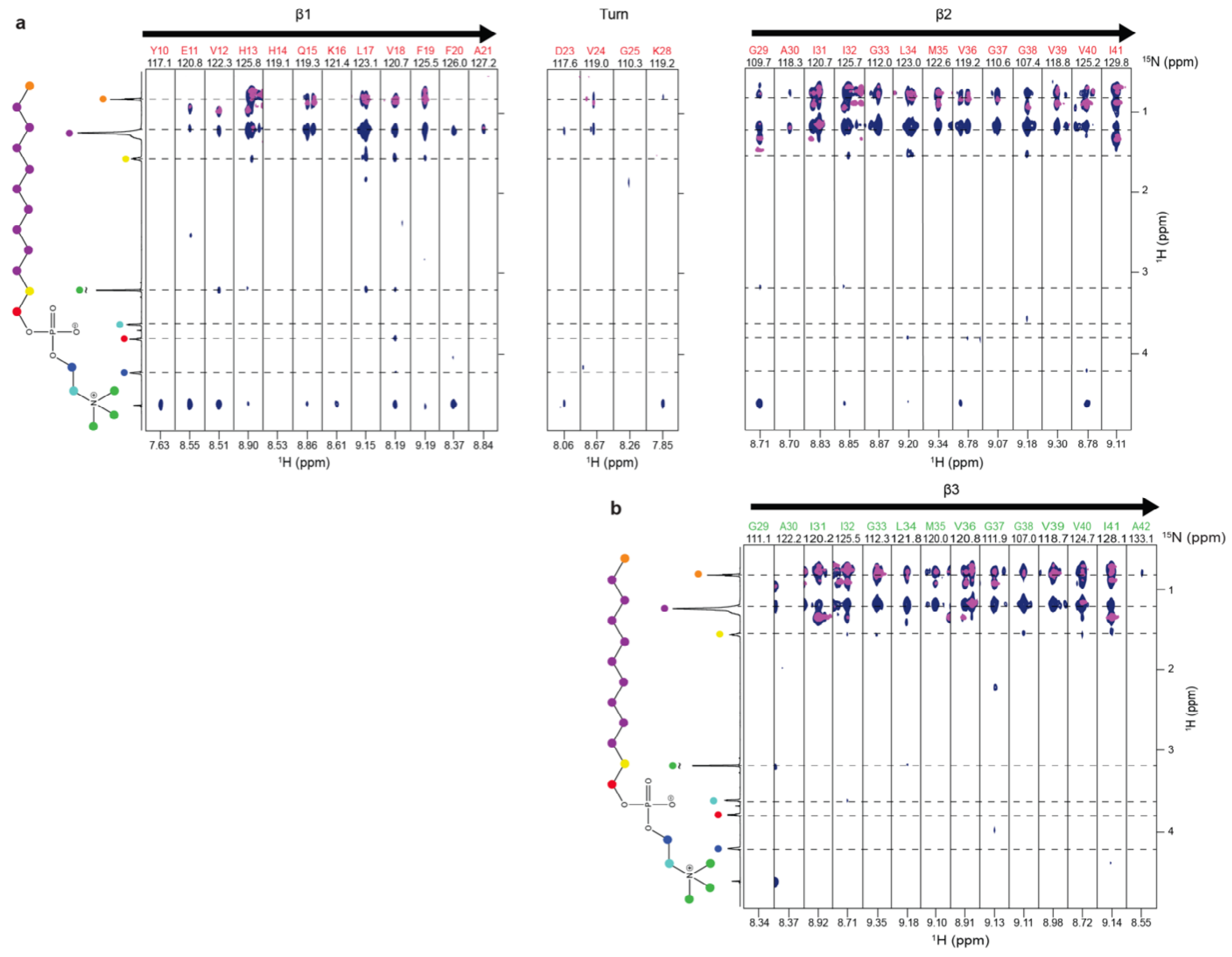

Supplementary Fig. 10. Strips of 3D ^{15}N-resolved [^{1}H,^{1}H]-NOESY spectrum. Spectrum was measured using a uniform ^{2}H,^{15}N, AILV-^{13}CH$_3$ Aβ(1-42) tetramer sample in deuterated DPC micelles (pink) and protonated DPC micelles (blue). The strips were taken at the ^{15}N chemical shifts of the residues indicated at the top and are centered around the respective amide proton chemical shifts for residues assigned to (**a**) the red Aβ(1-42) subunit and (**b**) the green Aβ(1-42) subunit. On the left side of the strips, one dimensional ^{1}H NMR spectrum of DPC, measured with the same sample as the 3D ^{15}N-resolved [^{1}H,^{1}H]-NOESY and the chemical structure of DPC. The CHn moieties of interest in this study are color-coded with magenta circles to indicate the CHn groups of the hydrophobic tails, and with green circles to identify the polar head methyls of DPC. Above the strips, arrows indicate β-strands.

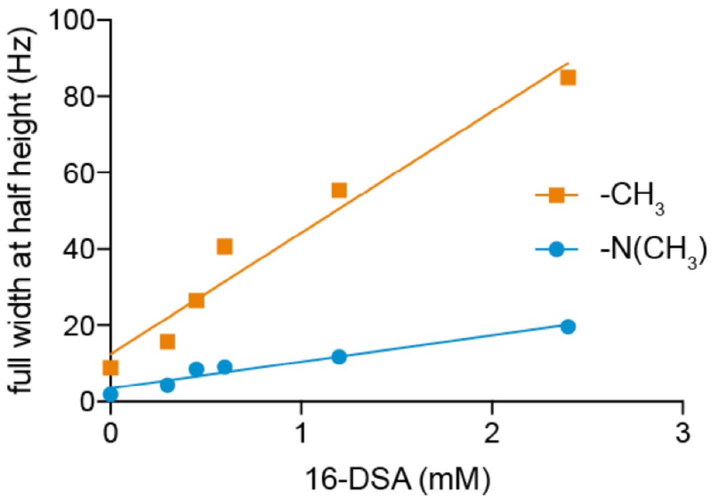

Supplementary Fig. 11. 16-DSA is properly inserted into DPC micelles. Line widths $\Delta\nu_{1/2}$ (full width at half weight) of DPC resonance in the 1D ^{1}H NMR spectra plotted against the concentration of 16-DSA. The data for the –CH3 resonance at 0.85 ppm (orange) and the –N(CH_3)$_3$ group at 3.26 ppm (blue) are shown. The ε values obtained from linear fits are indicated.

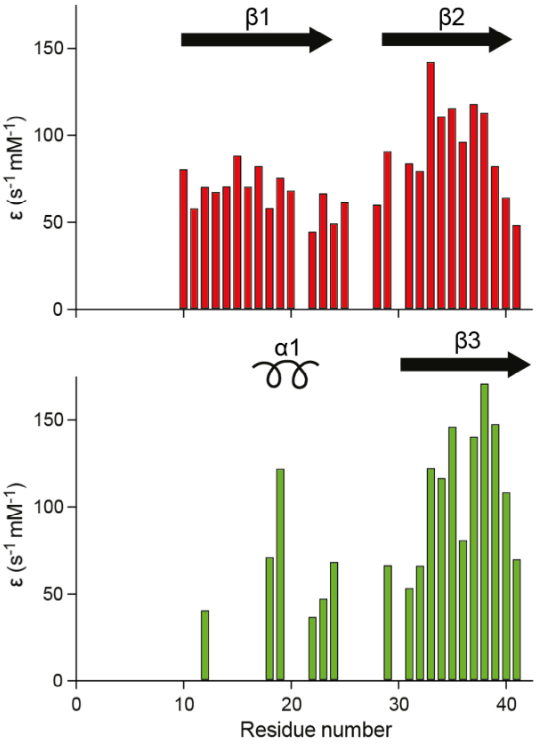

Supplementary Fig. 12. Effect of 16-DSA on backbone amide protons of the Aβ(1-42) tetramer. Paramagnetic relaxation enhancement, ε, vs. residue number for the red (top) and the green (bottom) Aβ(1-42) tetramer subunits estimated from the decay of the resonances in 2D [^{1}H,^{15}N]-HSQC spectra upon titration with 16-DSA. Secondary structural elements are shown on top with their corresponding number. Arrows indicate β-strands and helical symbols helices.

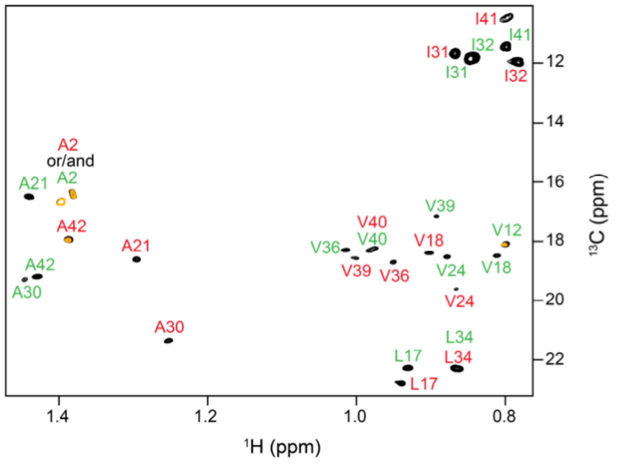

Supplementary Fig. 13. DPC micelles are arranged on both sides of the β-sheet core of the Aβ(1-42) tetramer. The 2D [^{1}H,^{13}C]-TROSY spectrum of Aβ(1-42) tetramer prepared using selectively ^{13}C methyl-protonated AILV and otherwise uniform ^{2}H,^{15}N Aβ(1-42) in DPC in the absence (black spectra) and in the presence of 0.6 mM 16-DSA (orange spectra). All signals corresponding to methyl groups located at either face of the β-sheet core disappeared while those located in the flexible N-termini remained, suggesting that the two faces of the six-stranded β-sheet core were in contact with the hydrophobic tail of DPC molecules.

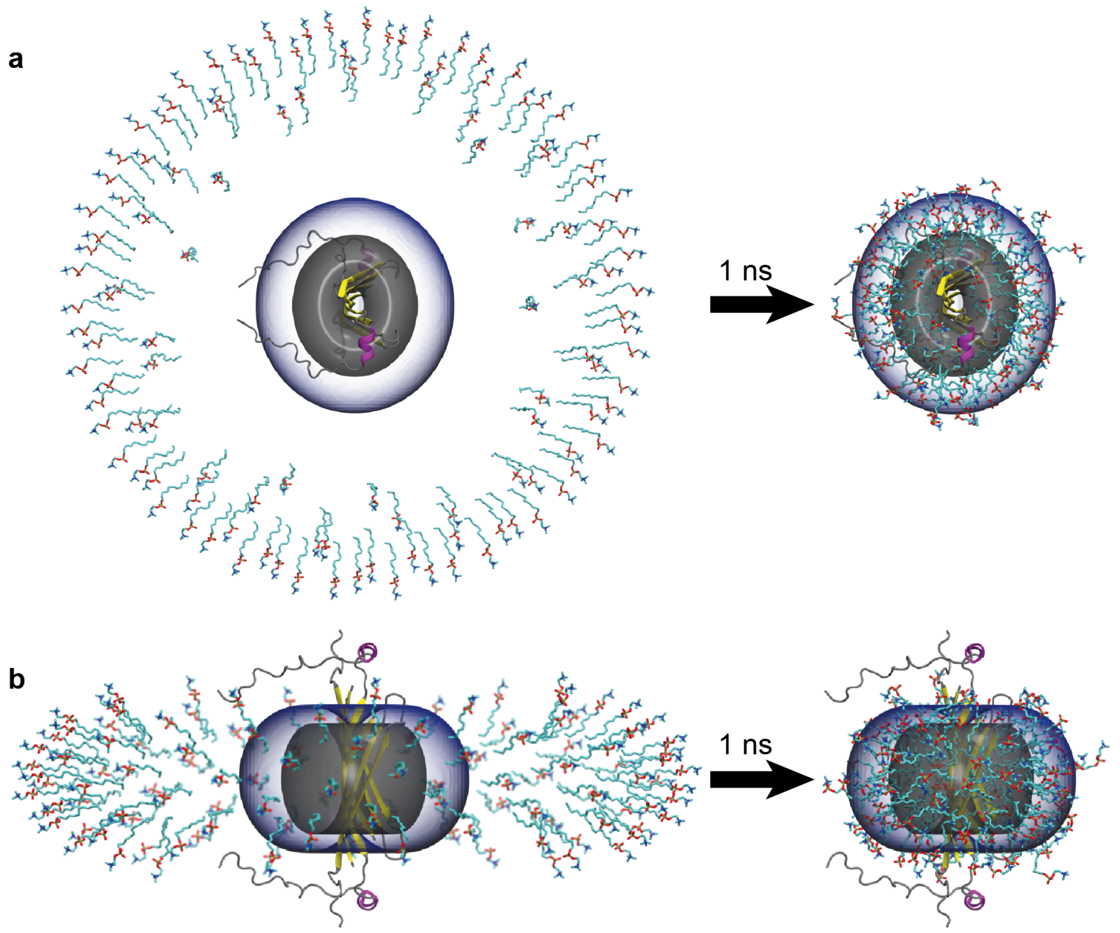

Supplementary Fig. 14. SimShape accelerated detergent assembly applied to Aβ(1-42) tetramer. Detergent molecules were initially placed far away from the protein assembly into the toroid-shaped potentials and left in an implicit solvent simulation with attractive grid potentials over the course of 1 ns, shown from top **(a)** and side **(b)**.

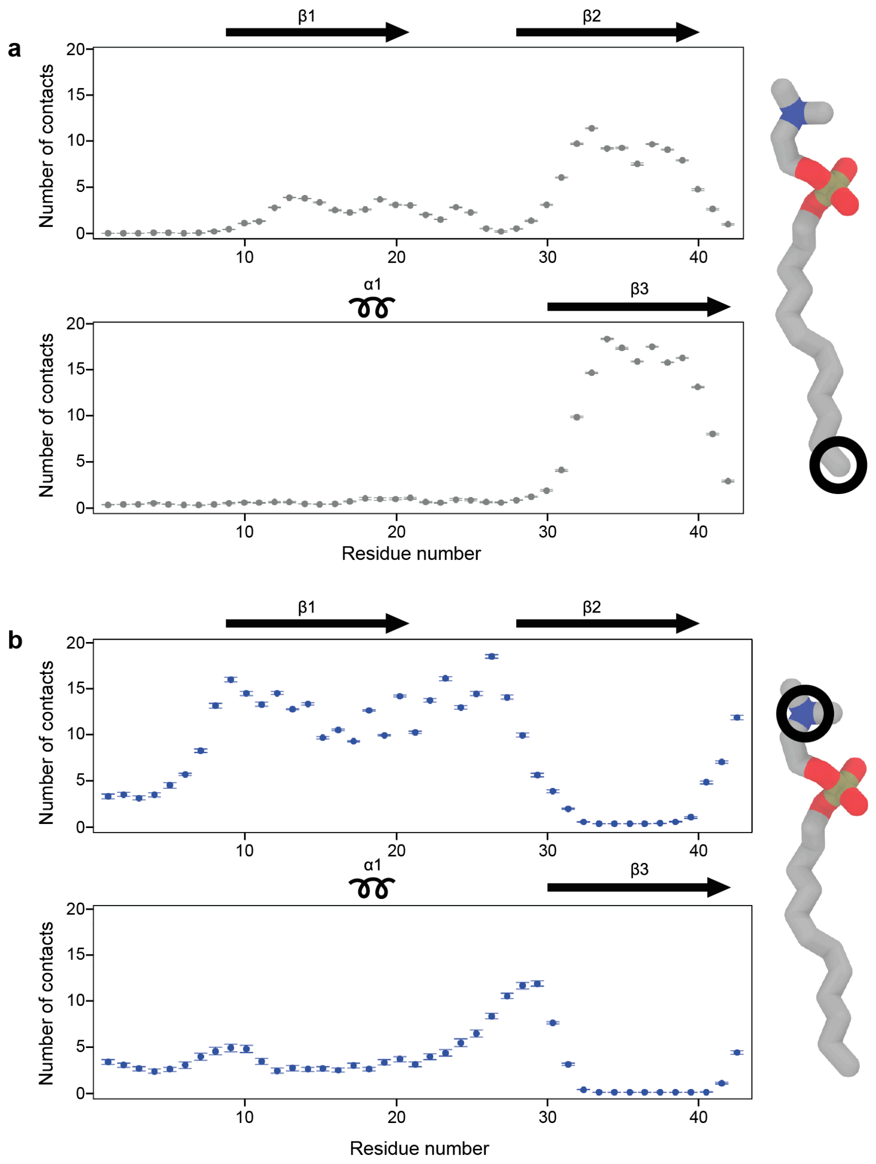

Supplementary Fig. 15. Contacts between Aβ(1-42) tetramer backbone nitrogen atoms and DPC. The average number of per-residue contacts with **(a)** DPC terminal tail carbon atoms and **(b)** DPC headgroup nitrogen atoms, summed over symmetric chains. Values are reported as the mean over eight independent replicates +/- S.E.M. DPC molecules drawn on the right of each panel show the atom used to define each contact with a black circle. Secondary structural elements are shown at the top of each panel with their corresponding number. Arrows indicate β-strands and helical symbols helices.

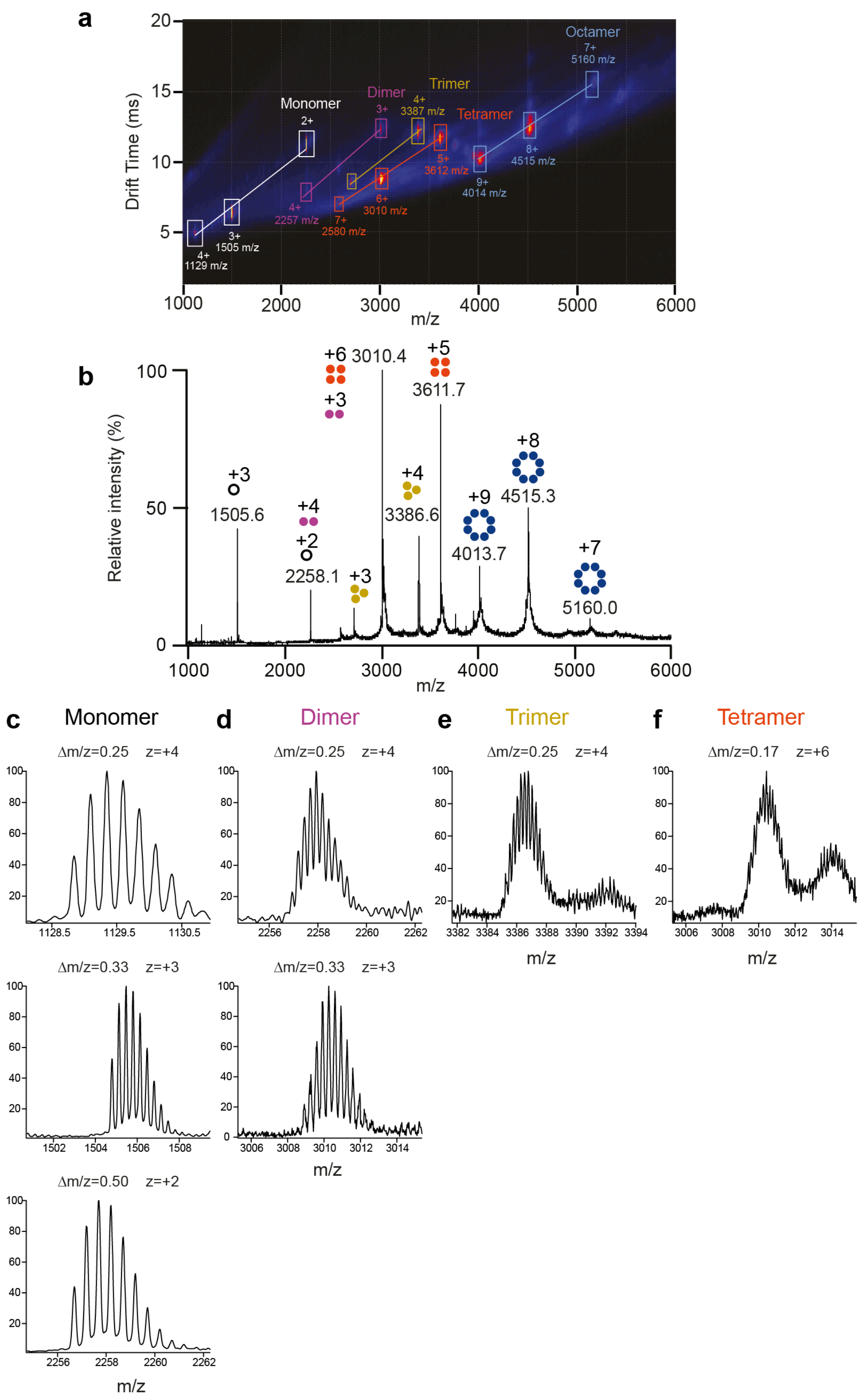

Supplementary Fig. 16. ESI-IM-MS analysis of βPFOs$_{\mathrm{LOW_A\beta(1\text{-}42)}}$. (**a**) ESI-IM-MS spectrum. Charge states corresponding to monomers, dimers, trimers, tetramers, and octamers are indicated, respectively, in white, yellow, pink, orange and blue. The number adjacent to each peak refers to the charge state of the ion. (**b**) Summed m/z spectrum showing the contribution of each oligomeric species to each peak in the

spectra. The charge states corresponding to monomers, dimers, trimers, tetramers, and octamers are indicated with schematic drawings and labeled, respectively, in white, pink, yellow, orange and blue. Associated m/z spectrum obtained for mobility peaks associated to (**c**) +4, +3, and +2 charge states of the monomer, (**d**) +4 and +3 charge states of the dimer, (**e**) +4 of the trimer and (**f**) +6 of the tetramer.

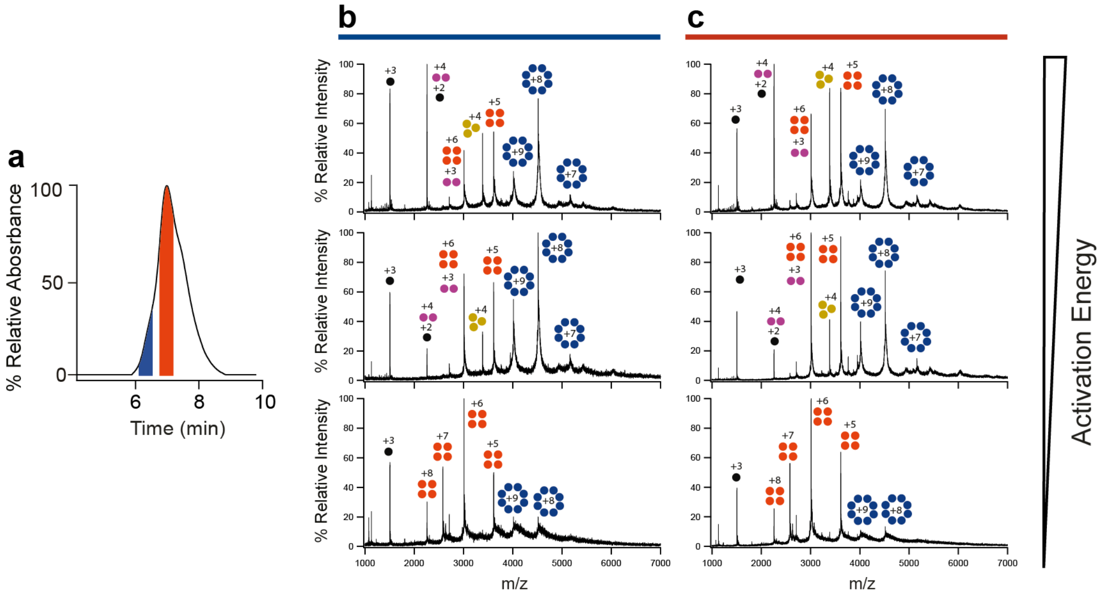

Supplementary Fig. 17. Effect of increasing the activation energy of βPFO$_{LOW_A\beta(1-42)}$ sample by SEC/IM-MS. (**a**) SEC chromatogram obtained with a column equilibrated in C8E5. The mass spectra extracted from the blue and orange SEC peaks and obtained at three activation energies are shown, respectively, with a (**b**) blue and (**c**) orange line on top of them. The charge states corresponding to monomers, dimers, trimers, tetramers, and octamers are indicated with schematic drawings and labeled, respectively, in black, pink, yellow, orange and blue.

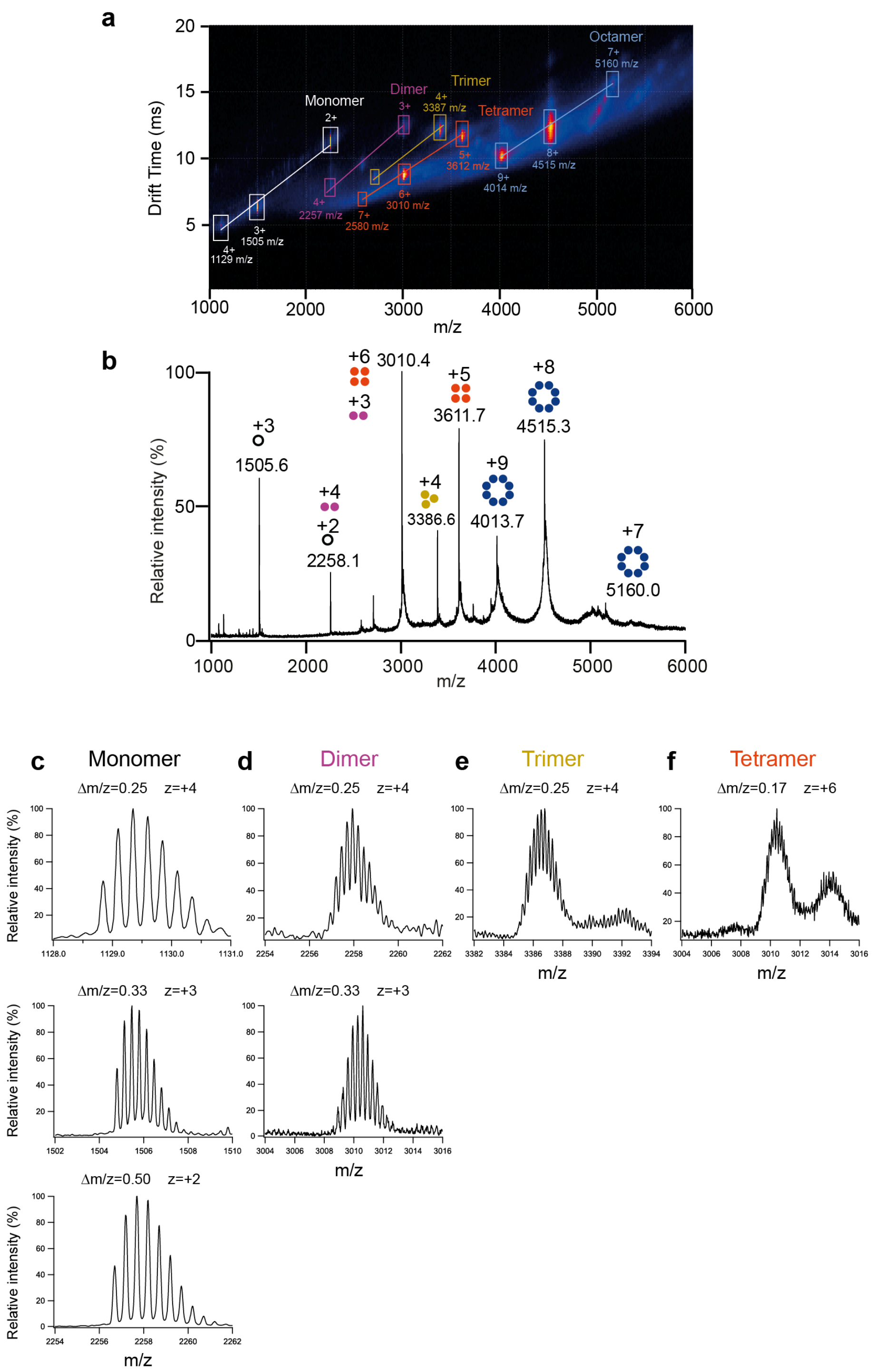

Supplementary Fig. 18. ESI-IM-MS analysis of βPFOs$_{HIGH_A\beta(1\text{-}42)}$. (**a**) ESI-IM-MS spectrum. Charge states corresponding to monomers, dimers, trimers, tetramers, and octamers are indicated, respectively, in white, yellow, pink, orange and blue. The number adjacent to each peak refers to the charge state of the ion. (**b**) Summed m/z spectrum showing the contribution of each oligomeric species to each peak in the

spectra. The charge states corresponding to monomers, dimers, trimers, tetramers, and octamers are indicated with schematic drawings and labeled, respectively, in white, pink, yellow, orange and blue. Associated m/z spectrum obtained for mobility peaks associated with (**c**) +4, +3, and +2 charge states of the monomer, (**d**) +4 and +3 charge states of the dimer, (**e**) +4 of the trimer and (**f**) +6 of the tetramer.

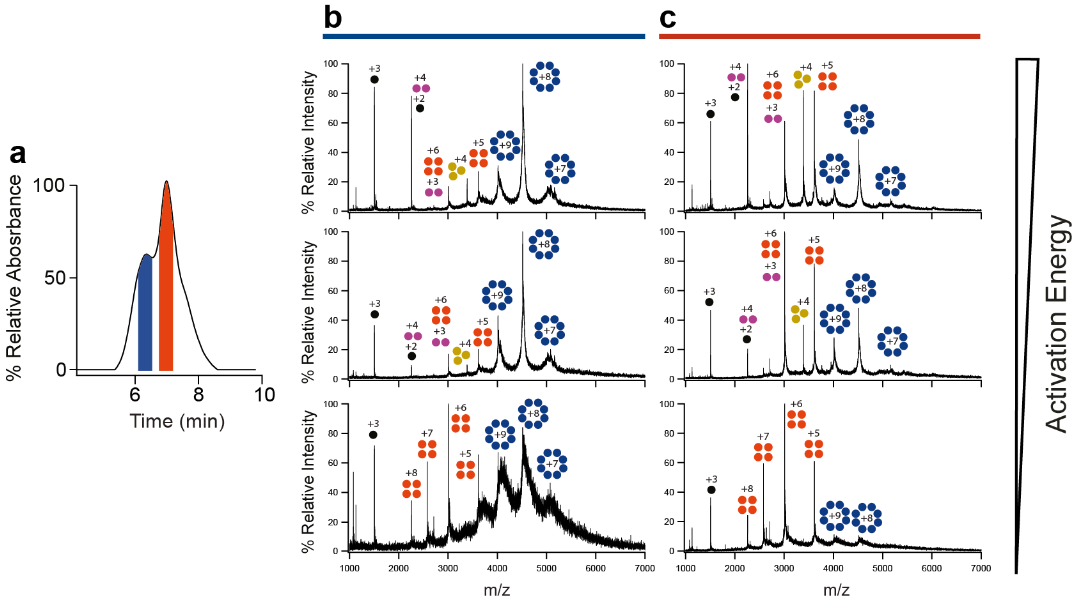

Supplementary Fig. 19. Effect of increasing the activation energy of βPFOs$_{HIGH_Aβ(1-42)}$, by SEC/IM-MS. (**a**) SEC chromatogram obtained with a column equilibrated in C8E5. The mass spectra extracted from the blue and orange SEC peaks and obtained at three activation energies are shown, respectively, with a (**b**) blue and (**c**) orange line on top of them. The charge states corresponding to monomers, dimers, trimers, tetramers, and octamers are indicated with schematic drawings and labeled, respectively, in black, pink, yellow, orange and blue.

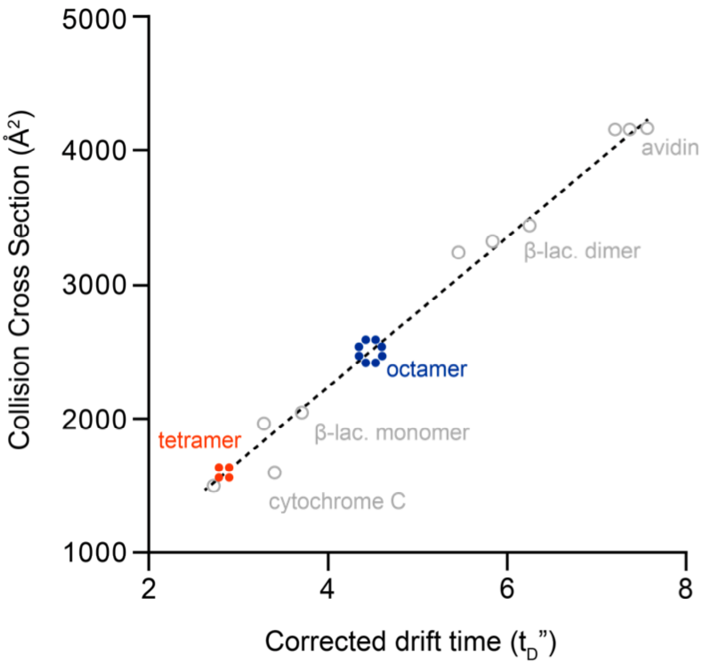

Supplementary Fig. 20. CCS calibration curve showing calibrants and Aβ(1-42) tetramer and octamer values. The graph was constructed combining data from cytochrome C, β-lactoglobulin monomer and dimer, and avidin displayed as CCS and corrected drift time. The regression line is represented as a dashed line (R^2 = 0.98). The interpolated CCS according to experimental corrected drift time measurements for Aβ(1-42) tetramer and octamer are shown as orange and dark blue representations, respectively.

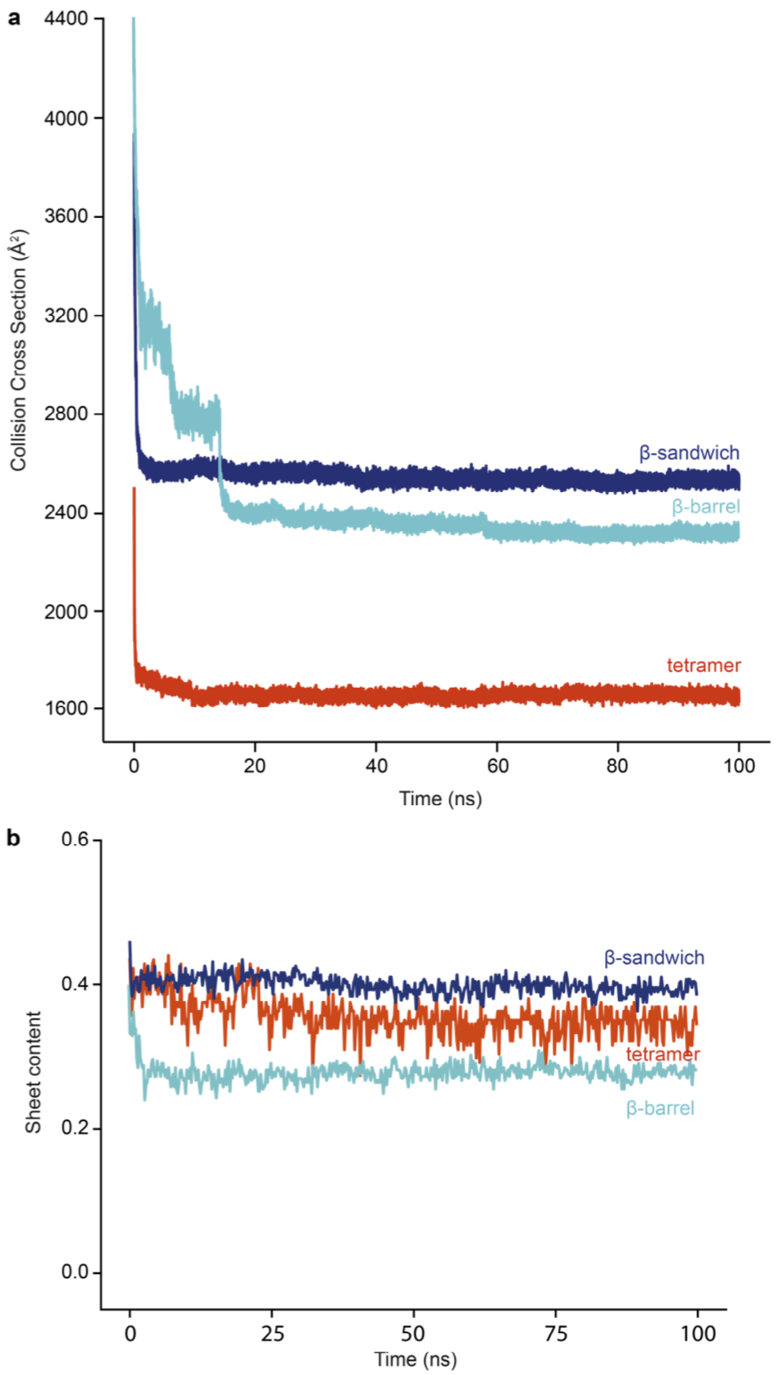

Supplementary Fig. 21. Gas phase evolution of theoretical CCS and β-sheet content of Aβ(1-42) tetramer and octamer structures. (**a**) Evolution of CCS and (**b**) β-sheet content for the +6 charge state of Aβ(1-42) tetramer structure (orange) and the +8 charge state of the two proposed octamer models: β-barrel (light blue) and β-sandwich structure (dark blue) along simulation time in vacuo.

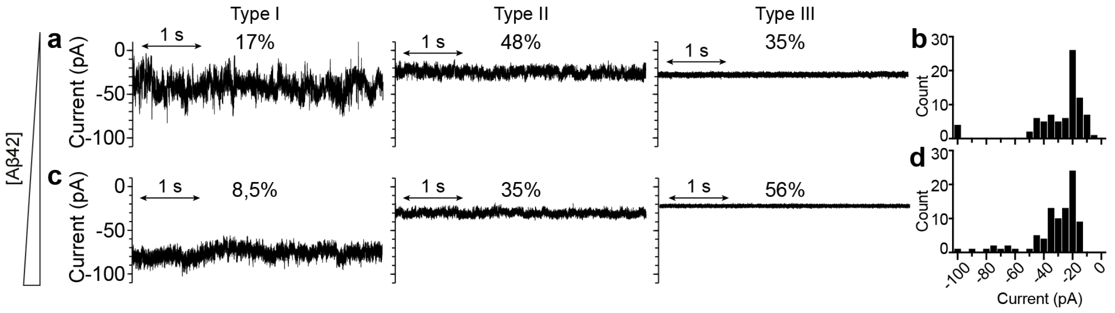

Supplementary Fig. 22. βPFOs$_{Aβ(1\text{-}42)}$ incorporate into lipid bilayers as pores. Typical current traces and relative abundance (shown on top of the current traces as percentages) for type 1, 2, and 3 pores observed for (**a**) βPFOs$_{LOW_Aβ(1\text{-}42)}$ and (**c**) βPFOs$_{HIGH_Aβ(1\text{-}42)}$. Electrical recordings were carried out on diphytanoyl-sn-glycero-3-phosphocholine planar lipid bilayers. All point histogram for type 2 and type 3 for (**b**) βPFOs$_{LOW_Aβ(1\text{-}42)}$ and (**d**) βPFOs$_{HIGH_Aβ(1\text{-}42)}$ under an applied potential of -100 mV. Controls were carried out to establish that the concentration of the detergent micelles present in the samples did not affect the stability of the bilayer.

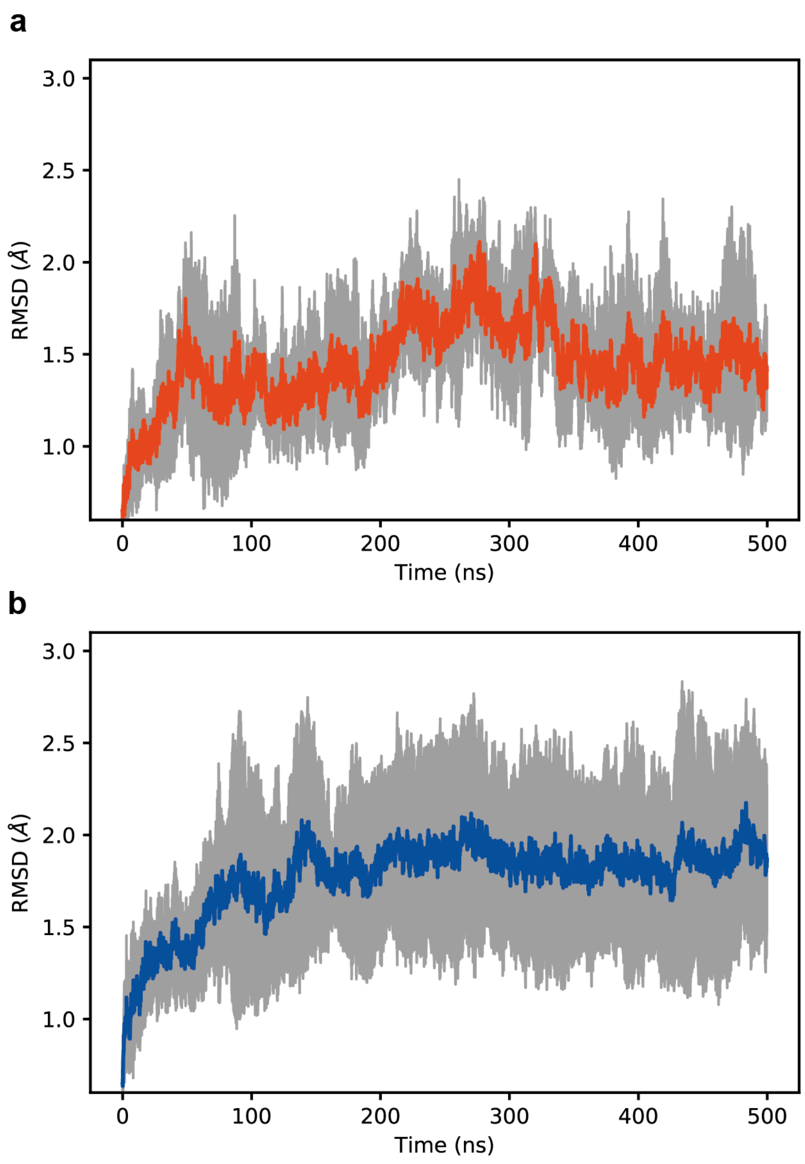

Supplementary Fig. 23. Time dependence of the average root mean square deviations (RMSD) of backbone atoms. (**a**) Aβ(1-42) tetramer structure (orange) and (**b**) β-sandwich octamer structure (dark blue) along the 500 ns simulation time. The standard deviation among three replicates is shown in grey.

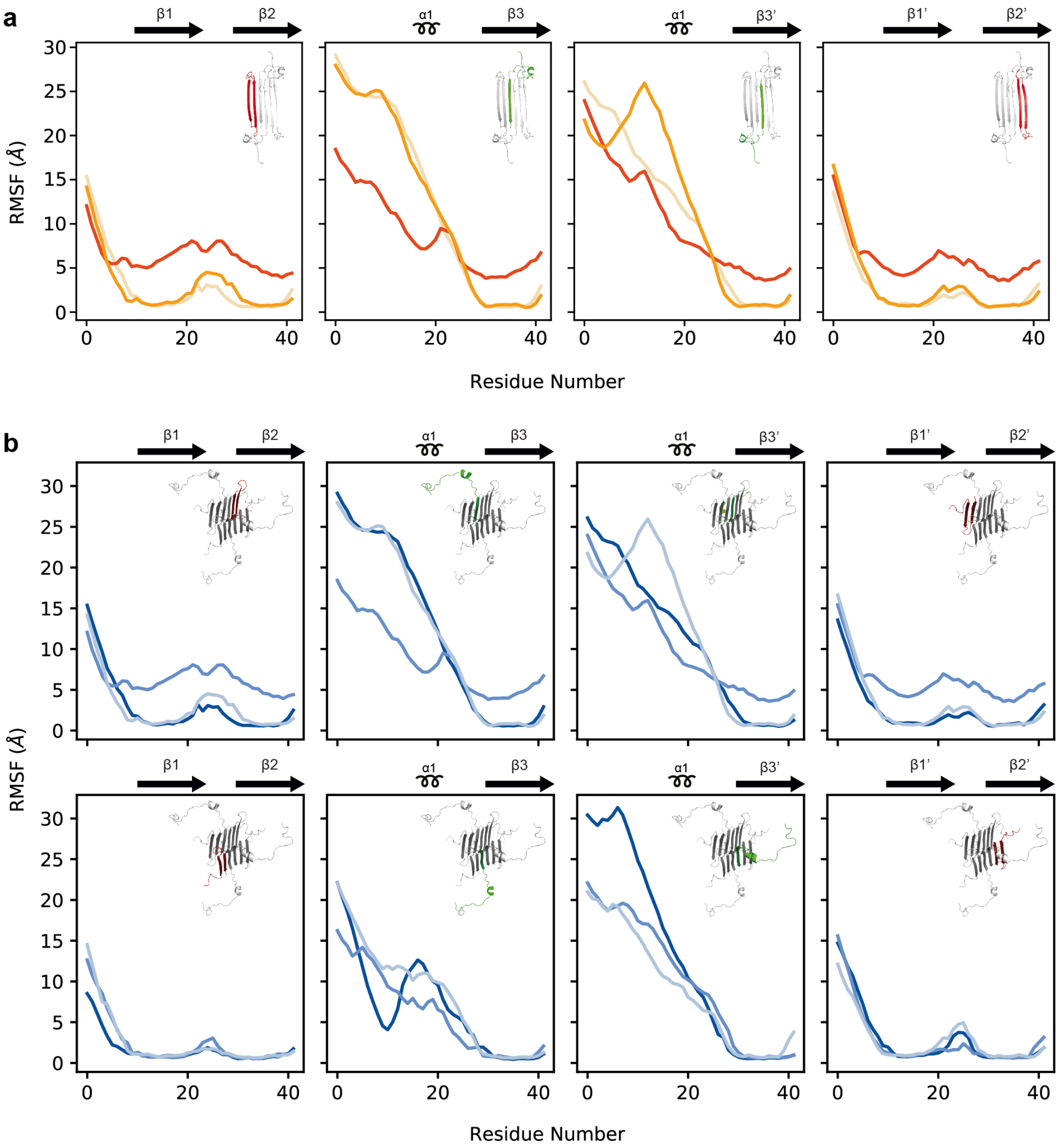

Supplementary Fig. 24. Root mean square fluctuations (RMSF) versus residue number. RMSF was calculated from three replicates for (**a**) the four Aβ(1-42) subunits comprising the tetramer structure and (**b**) the eight Aβ(1-42) subunits comprising the β-sandwich octamer structure. In each panel, a representation of the type of Aβ(1-42) subunit analysed, either red or green, is shown. The presence of β-strands and helices within each Aβ(1-42) subunit are indicated as arrows and helical symbols, respectively.

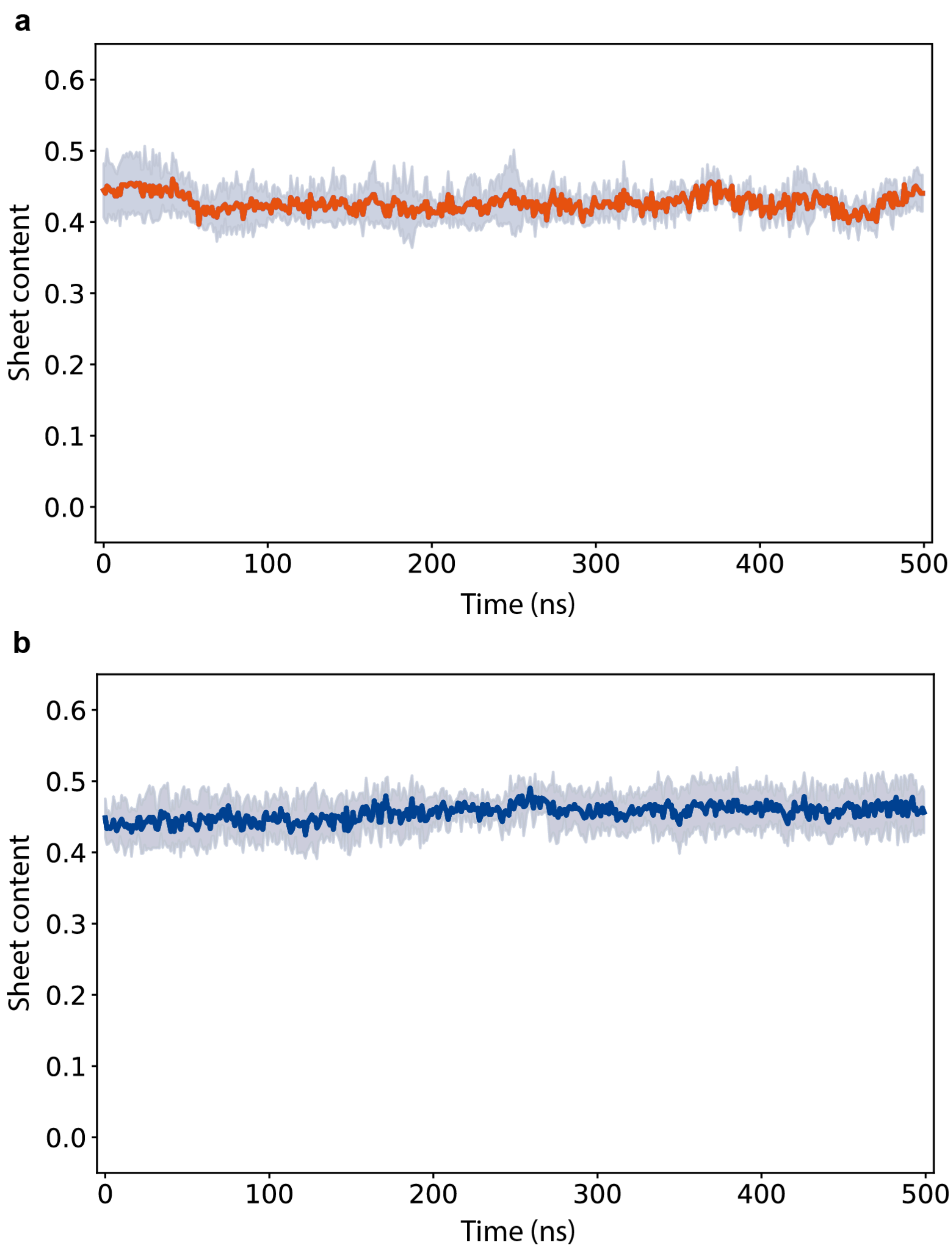

Supplementary Fig. 25. Time dependence of the average variation of β-sheet content. (**a**) Aβ(1-42) tetramer structure (orange) and (**b**) Aβ(1-42) β-sandwich octamer structure (dark blue) during the 500 ns MD simulation. The standard deviation among three replicates is shown in grey.

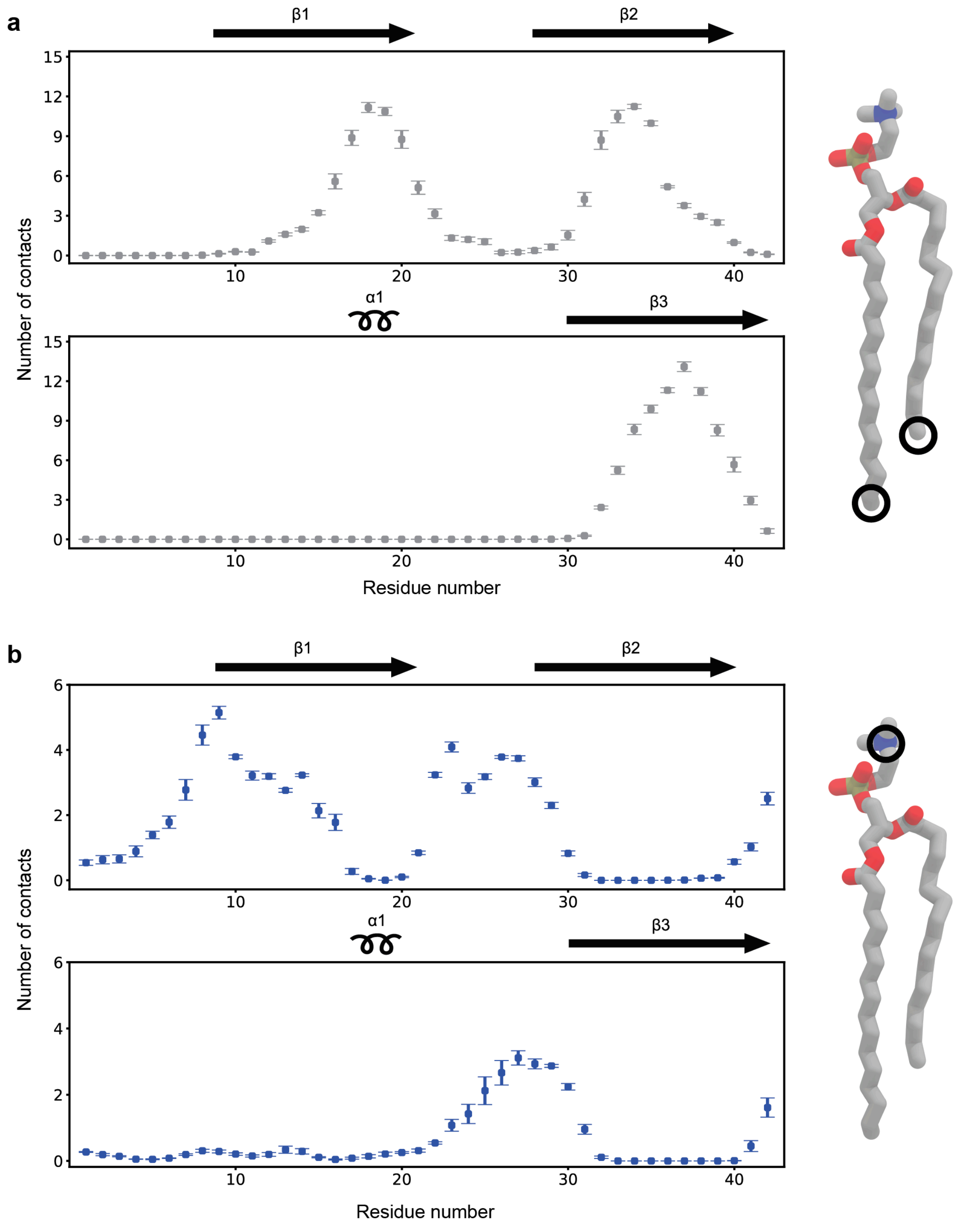

Supplementary Fig. 26. Contacts between tetramer backbone nitrogen atoms and DPPC. The average number of per-residue contacts with DPPC hydrophobic tail atoms (**a**) and DPPC headgroup nitrogen atoms (**b**), summed over symmetric chains. Values are reported as the mean over three independent replicates +/- S.E.M. DPPC molecules (right) mark the atom used to define each contact with a black circle.

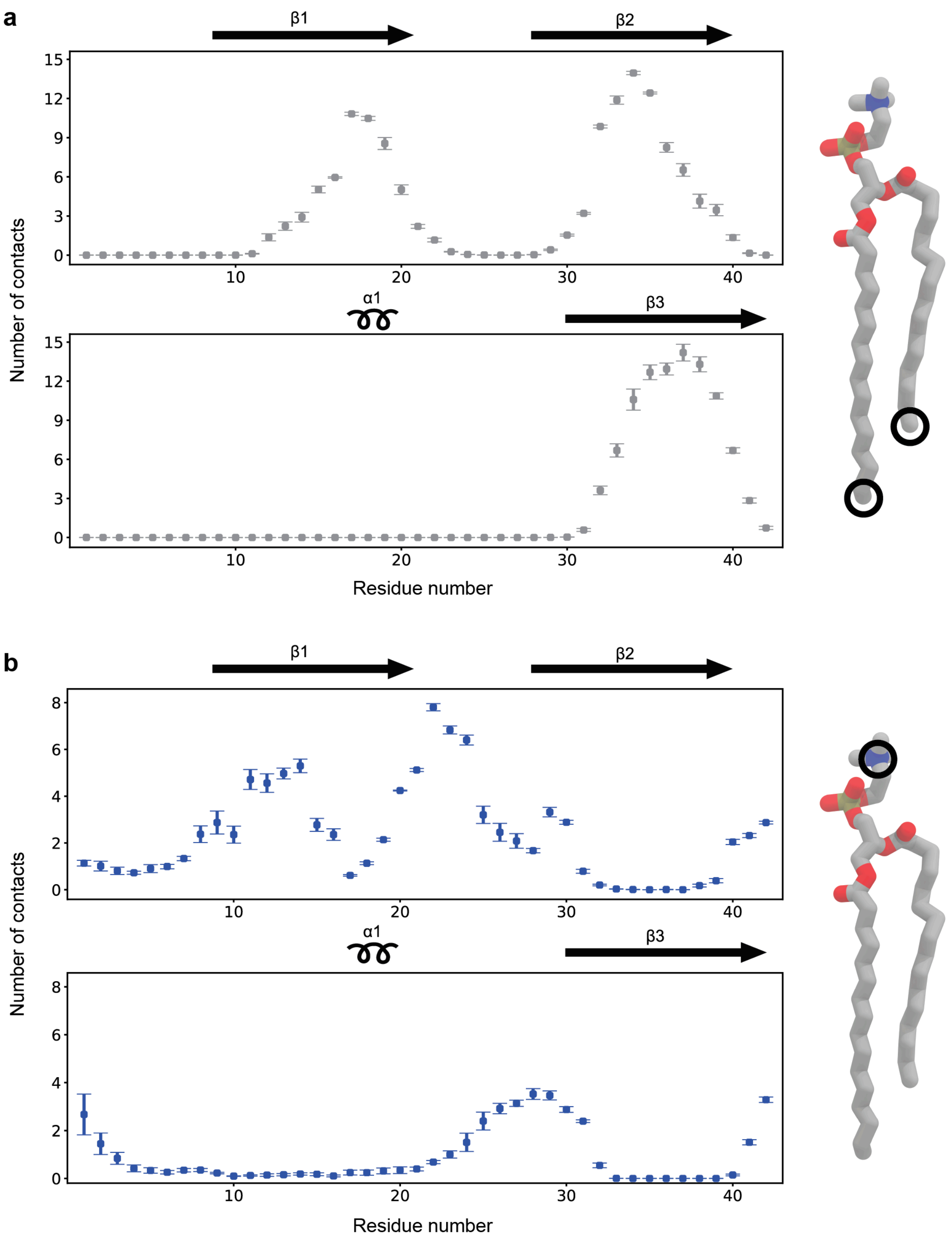

Supplementary Fig. 27. Contacts between octamer backbone nitrogen atoms and DPPC. The average number of per-residue contacts with DPPC hydrophobic tail atoms (**a**) and DPPC headgroup nitrogen atoms (**b**), summed over symmetric chains. Values are reported as the mean over three independent replicates +/- S.E.M. DPPC molecules (right) mark the atom used to define each contact with a black circle.

Supplementary Tables

Supplementary Table 1: Number of unique NOE-derived distance restraints used for structure calculation of Aβ(1-42) tetramers (per dimer unit).

NOE distance restraints	all	HN-HN	HN-Met	Met-Met
total	**157**	45	87	25
intra-monomer	**107**	35	60	12
intra-residual (|i-j| = 0)	**22**	0	22	0
sequential (|i-j| = 1)	**44**	24	20	0
medium-range (2 ≤ |i-j| < 5)	**14**	3	5	6
long-range (|i-j| ≥ 5)	**26**	8	12	6
ambiguous	**1**	0	1	0
inter-monomer	**30**	7	15	8
inter-dimer	**13**	3	5	5
ambiguous	**7**	0	7	0

Supplementary Table 2: Structure and restraints statistics for the Aβ(1-42) tetramer

**Number of restraints (per dimer)**	
NOE distance restraints	
Intra-momomer	
Intra-residue (|i-j| = 0)	22
Sequential (|i-j| = 1)	44
Medium-range (2 ≤ |i-j| < 5)	14
Long-range (|i-j| ≥ 5)	26
Ambiguous	1
Total	*107*
Inter-monomer	30
Inter-dimer	13
Ambiguous	7
Total	*157*
Dihedral angle restraints (ϕ/ψ)	82 (41/41)
Hydrogen-bond restraints*	
Intra-momomer	24
Inter-momomer	28
Inter-dimer	12
Total	*64*

**Restraints statistics[a]**	
RMS of distance violations	
NOE restraints	0.007 ± 0.002 Å
H-bonds restraints	0.001 ± 0.001 Å
Distance violations (count per conformer)	
> 0.5 Å	0
> 0.3 Å	0
> 0.1 Å	0
RMS of dihedral violations	0.33 ± 0.11°
Dihedral violations > 5° (count per conformer)	0
**RMS from idealized covalent geometry**	
bonds	0.003 ± 0.001 Å
angles	0.376 ± 0.007°
impropers	0.898 ± 0.091°
**Structural quality[a]**	
Ramachandran statistics[b]	
Most favoured regions	76.0 ± 4.0 %
Allowed regions	23.7 ± 3.7 %
Disallowed regions	0.3 ± 0.6 %
Molprobity	
Clashscore	1.88 ± 0.64 (99th percentile)
Coordinates precision[c]	
Backbone atoms (core β-sheet[d], tetramer)	0.77 ± 0.27 Å
Heavy atoms (core β-sheet, tetramer)	1.34 ± 0.18 Å
Backbone atoms (core β-sheet, dimer)	0.54 ± 0.14 Å
Heavy atoms (core β-sheet, dimer)	1.19 ± 0.12 Å

*One hydrogen-bond is encoded with 2 restraints (HN…O and N…O)

[a] Average values and standard deviations over the 15 models

[b] Percentage of residues in the Ramachandran plot regions determined by PROCHECK[1]

[c] Average root mean square deviation (RMSD) over the 15 conformers with respect to the average structure.

[d] Residues β1-β2-β3-β1'-β2'-β3' (only β1-β2-β3 for dimer)

Supplementary Table 3: Theoretical and measured masses for the different Aβ(1-42) species detected in native mass spectrometry experiments. Values listed are averages of three individual measurements performed on three individual replicates. Source data are provided as Source Data file.

	Average measured mass (Da)	Theoretical mass (Da)	Error (ppm)
monomer	4.514,1 ± 0,1	4.514,0	6
dimer	9.027,8 ± 0,1	9.028,1	27
trimer	13.541,9 ± 0,1	13.542,1	20
tetramer	18.056,0 ± 0,1	18.056,2	8
octamer	36.114,4 ± 0,2	36.112,3	59

Supplementary Table 4: Theoretical and measured masses for the different Aβ(1-42) species detected in MALDI mass spectrometry experiments. Values listed are averages of three individual measurements performed on three individual replicates. Source data are provided as Source Data file.

	Average measured mass (Da)	Theoretical mass (Da)
monomer	4.403 ± 4 *	4.514,04
monomer XL	4.886 ± 21 **	4.514,04 ***
tetramer XL	19.636 ± 47 **	18.056,16 ***
octamer XL	38.973 ± 153 **	36.112,32 ***

High-Mass MALDI is a technique used to determine the molecular weight of a protein sample from 50 kDa up to 2 MDa. Therefore, since this technique improves transmission of ions of MW larger than 20 kDa, it is not accurate to measure molecular weights lower than 50 kDa. It is for this reason that the measured and theoretical mass errors were not calculated. Nevertheless, relative masses are of high confidence.

* Independent classical MALDI analysis performed in reflecton mode on Aβ(1-42) monomer, with a calibration suited for the monomer mass range, led to an average measured mass of 4.513,6 corresponding to an error of 97 ppm, which is in agreement with the expected error for MALDI-MS analysis of this molecular weight.

** Average measured masses for cross-linked (XL) species (monomer XL, tetramer XL, octamer XL) correspond to the mass of the XL Aβ(1-42) species including activated carboxylic acids that remain unreacted.

*** Theoretical masses for cross-linked (XL) species (monomer XL, tetramer XL, octamer XL) have been calculated as the intrinsic mass of the non XL Aβ(1-42) species without considering the losses of water due to the formation of XLs and activated carboxylic acids that remain unreacted.

Supplementary Table 5: Acquisition parameters of the NMR experiments carried out throughout this study

Experiment	D1	NS	NUS %	F3<br>AQ (ms) / SW (ppm)	F2<br>AQ (ms) / SW (ppm)	F1<br>AQ (ms) / SW (ppm)	NOE mix (s)	Exp time (h)
3D HNCA (pH 8.5)	1.5	8	25	71/18	40/32	25/46		93
3D HNCA (pH 9.5)	1.5	8	30	71/18	40/32	45/26		89
3D HNCACB (pH 8.5)	1.5	16	30	71/18	18/46	14/75		129
3D HNCACB (pH 9.5)	1.2	16	30	71/18	22/26	12/75		64
HNCO (pH 9.5)	1.5	64	50		63/40	79/47		18
3D HN-NH NOESY	0.25	16	25	70/6	30/3	37/26	0.08	94
3D (Hme)Cme([C] CA)CO	1.0	8	50	61/14	14/16	10/14		9
3D (Hme)Cme([C] CA)NH	1.0	16	50	61/14	22/25	11/16		22
3D Hme(Cme[C]CA) NH	1.0	16	50	61/14	22/25	18/2		17.5
3D (H)C-TOCSY-C-TOCSY-(C)H	1.25	8	30	57/20	8.6/66	11/16		19.5
3D Hm-CmHm NO-ESY	0.2	16	50	57/10	15/14	36/1	0.3	8.5
3D Cm- CmHm NO-ESY	0.3	64	65.1	57/10	15/14	15/14	0.2	66.5
3D Hm-NH NOESY	0.2	16	50	57/20	27/26	36/1	0.3	12.5
3D Cm-NH NOESY	0.2	16	50	57/20	27/26	15/14	0.3	17

Supporting References

1. Laskowski, R. A., MacArthur, M. W., Moss, D. S. & Thornton, J. M. PROCHECK: a program to check the stereochemical quality of protein structures. *J. Appl. Cryst* ***26,*** *283-291* (1993).
