# Supplementary material for: Aβ(1-42) tetramer and octamer structures reveal edge conductivity pores as a mechanism for membrane damage
Source: Nat Commun. 2020 Jun 15;11:3014. doi: 10.1038/s41467-020-16566-1 (PMC7296003; doi:10.1038/s41467-020-16566-1)
Supplement: Supplementary file 3 — Reporting Summary [file 41467_2020_16566_MOESM3_ESM.pdf]

nature research

Corresponding author(s): Natàlia Carulla, Ph.D.

Last updated by author(s): Mar 16, 2020

Reporting Summary

Nature Research wishes to improve the reproducibility of the work that we publish. This form provides structure for consistency and transparency in reporting. For further information on Nature Research policies, see Authors & Referees and the Editorial Policy Checklist .

Statistics

For all statistical analyses, confirm that the following items are present in the figure legend, table legend, main text, or Methods section.

n/a	Confirmed	
☐	☒	The exact sample size (*n*) for each experimental group/condition, given as a discrete number and unit of measurement
☐	☒	A statement on whether measurements were taken from distinct samples or whether the same sample was measured repeatedly
☒	☐	The statistical test(s) used AND whether they are one- or two-sided<br>*Only common tests should be described solely by name; describe more complex techniques in the Methods section.*
☒	☐	A description of all covariates tested
☒	☐	A description of any assumptions or corrections, such as tests of normality and adjustment for multiple comparisons
☐	☒	A full description of the statistical parameters including central tendency (e.g. means) or other basic estimates (e.g. regression coefficient) AND variation (e.g. standard deviation) or associated estimates of uncertainty (e.g. confidence intervals)
☒	☐	For null hypothesis testing, the test statistic (e.g. *F*, *t*, *r*) with confidence intervals, effect sizes, degrees of freedom and *P* value noted<br>*Give P values as exact values whenever suitable.*
☒	☐	For Bayesian analysis, information on the choice of priors and Markov chain Monte Carlo settings
☒	☐	For hierarchical and complex designs, identification of the appropriate level for tests and full reporting of outcomes
☒	☐	Estimates of effect sizes (e.g. Cohen's *d*, Pearson's *r*), indicating how they were calculated

Our web collection on statistics for biologists contains articles on many of the points above.

Software and code

Policy information about availability of computer code

Data collection	For protein purification: Unicorn v7.0 (GE Healthcare)<br>For NMR experiments: TopSpin 3.5 (Bruker)<br>For simulations: SimShape, NAMD 2.13, CHARMM-GUI 2.0<br>For IM/MS experiments: MassLynx 4.1 (Waters)<br>For XL/MS experiments: FlexAnalysis 3.4 (Bruker)
Data analysis	For protein purification: Unicorn v7.0 (GE Healthcare)<br>For NMR experiments: TopSpin 3.5, MDDNMR 2.5, and CCPNmr Analysis 2.4.2<br>For NMR structure calculation: ARIA 2.3.2/CNS 1.21 and TALOS-N 4.21, PROCHECK 3.5.4, WHATIF 8.3, and MolProbity 3.19<br>For simulations: VMD 1.9.4a37, MDTraj 1.9.3<br>For IM/MS experiments: MassLynx 4.1 (Waters), DriftScope 2.4 (Waters)<br>For CCS calculations: Impact 1.0<br>For XL MS experiments: flexAnalysis 3.4 (Bruker)<br>For electrical recordings: Clampfit 10

For manuscripts utilizing custom algorithms or software that are central to the research but not yet described in published literature, software must be made available to editors/reviewers. We strongly encourage code deposition in a community repository (e.g. GitHub). See the Nature Research guidelines for submitting code & software for further information.

Data

Policy information about availability of data

All manuscripts must include a data availability statement. This statement should provide the following information, where applicable:

- Accession codes, unique identifiers, or web links for publicly available datasets
- A list of figures that have associated raw data
- A description of any restrictions on data availability

Accession codes for deposited data: Coordinates have been deposited in the Protein Data Bank under accession number 6RHY, and chemical shifts have been deposited in the Biological Magnetic Resonance Bank under entry 34396.
The authors declare that the main data supporting the findings of this study is available within the Article and the Supplementary Information or from the authors upon request. The source data underlying Figs. 1a,b, 2c,d, 3a-f, 4a,b,e,f and Supplementary Tables S3 and S4 are provided as a Source Data file.

Field-specific reporting

Please select the one below that is the best fit for your research. If you are not sure, read the appropriate sections before making your selection.

[x] Life sciences [ ] Behavioural & social sciences [ ] Ecological, evolutionary & environmental sciences

For a reference copy of the document with all sections, see nature.com/documents/nr-reporting-summary-flat.pdf

Life sciences study design

All studies must disclose on these points even when the disclosure is negative.

Sample size	No sample-size calculation was performed. Sample size was chosen based on previous experience and standards in the field. At least three independent samples were analyzed for each group/condition.
Data exclusions	No data were excluded from the analysis.
Replication	We confirm that experiments were replicated and all attempts at replication were successful.
Randomization	We analyzed our samples systematically rendering randomization not necessary in our experiments.
Blinding	Our experiments did not involve subjective trials rendering blindings not necessary in our experiments.

Reporting for specific materials, systems and methods

We require information from authors about some types of materials, experimental systems and methods used in many studies. Here, indicate whether each material, system or method listed is relevant to your study. If you are not sure if a list item applies to your research, read the appropriate section before selecting a response.

Materials & experimental systems

n/a	Involved in the study	
[x]	[ ]	Antibodies
[x]	[ ]	Eukaryotic cell lines
[x]	[ ]	Palaeontology
[x]	[ ]	Animals and other organisms
[x]	[ ]	Human research participants
[x]	[ ]	Clinical data

Methods

n/a	Involved in the study	
[x]	[ ]	ChIP-seq
[x]	[ ]	Flow cytometry
[x]	[ ]	MRI-based neuroimaging
